# High Intensity Focused Ultrasound versus Brachytherapy for the Treatment of Localized Prostate Cancer: A Matched-Pair Analysis

**DOI:** 10.1155/2015/350324

**Published:** 2015-08-19

**Authors:** Fouad Aoun, Ksenija Limani, Alexandre Peltier, Quentin Marcelis, Marc Zanaty, Alexandre Chamoun, Marc Vanden Bossche, Thierry Roumeguère, Roland van Velthoven

**Affiliations:** ^1^Department of Urology, Jules Bordet Institute, Université Libre de Bruxelles, 1000 Brussels, Belgium; ^2^Department of Urology, Erasme Hospital, Université Libre de Bruxelles, 1070 Brussels, Belgium

## Abstract

*Purpose*. To evaluate postoperative morbidity and long term oncologic and functional outcomes of high intensity focused ultrasound (HIFU) compared to brachytherapy for the treatment of localized prostate cancer.* Material and Methods*. Patients treated by brachytherapy were matched 1 : 1 with patients who underwent HIFU. Differences in postoperative complications across the two groups were assessed using Wilcoxon's rank-sum or *χ* ^2^ test. Kaplan-Meier curves, log-rank tests, and Cox regression models were constructed to assess differences in survival rates between the two groups.* Results*. Brachytherapy was significantly associated with lower voiding LUTS and less frequent acute urinary retention (*p* < 0.05). Median oncologic follow-up was 83 months (13–123 months) in the HIFU cohort and 44 months (13–89 months) in the brachytherapy cohort. Median time to achieve PSA nadir was statistically shorter in the HIFU. Biochemical recurrence-free survival rate was significantly higher in the brachytherapy cohort compared to HIFU cohort (68.5% versus 53%, *p* < 0.05). No statistically significant difference in metastasis-free, cancer specific, and overall survivals was observed between the two groups.* Conclusion*. HIFU and brachytherapy are safe with no significant difference in cancer specific survival on long term oncologic follow-up. Nonetheless, a randomized controlled trial is needed to confirm these results.

## 1. Introduction

In the last two decades, transperineal low dose rate (LDR) brachytherapy emerged as a therapeutic option for patients with organ confined prostate cancer. This technique was supported by technical advances in transrectal ultrasound (TRUS), advent of template guidance, and improved dosimetry [1]. In 2012, the American Brachytherapy Society (ABS) provided an updated consensus guideline on patient selection, workup, treatment, postimplant dosimetry, and follow-up [2]. The panel recommended prostate brachytherapy as a monotherapy for low risk organ confined prostate cancer patients and some patients with intermediate risk disease [2]. The technique is safe and effective but carries a nonnegligible risk of severe toxicities to the urethra, bladder, neurovascular bundles, and rectum because of their anatomic proximity to the prostate and the high dose intensity close to the radiation source [3]. In addition, the rapid decline in radiation dose can lead to suboptimal control outside the planned area of treatment [4]. Available evidence on oncologic outcomes is based on case series with only one prospective randomized trial comparing brachytherapy to other primary treatment options for organ confined prostate cancer [5]. These facts have contributed to the development and application of new minimally invasive approaches to organ confined prostate cancer. Among these therapies, high intensity focused ultrasound (HIFU) emerged as a valid mini-invasive therapy for organ confined prostate cancer, using focused ultrasound to generate areas of intense heat to induce tissue necrosis. The ability of HIFU to achieve thermoablation of prostatic lesion was proven on MRI imaging and histologically on posttreatment biopsies and on operative specimens [6–9]. Oncologic outcomes were first reported in mid-1990 and subsequently the use of HIFU therapy has expanded [10, 11]. Different case series have been published reporting safety and efficacy of HIFU as well as favorable perioperative and oncologic results. Recently, we published long term results of a cohort of 110 consecutive patients with organ confined prostate cancer primarily treated with whole gland HIFU [12]. At ten years of follow-up, we estimated a biochemical recurrence-free survival (BRFS) rate, an overall survival (OS) rate, and a cancer specific survival (CSS) rate of 40%, 72%, and 90%, respectively. Nonetheless, the European Association of Urology (EAU), the American Urological Association (AUA), and the National Comprehensive Cancer Network (NCCN) do not recommend the routine use of HIFU in the primary treatment of prostate cancer given the absence of prospective randomized controlled trials comparing HIFU with conventional treatment options and the paucity of long term oncologic follow-up data. The aim of this study is to evaluate peri- and postoperative morbidity and long term oncologic and functional outcomes of whole gland HIFU compared with brachytherapy. We thus performed a matched-pair analysis controlling for clinical and pathologic variables comparing patients treated by HIFU and brachytherapy during the same period.

## 2. Materials and Methods

Patients scheduled to undergo brachytherapy or HIFU for organ confined prostate cancer in our two academic hospitals were prospectively enrolled between September 2001 and December 2012. Pooled prospectively collected data were retrospectively analyzed. Institutional review board approval was obtained from the two centers. Inclusion criteria for the two groups of patients were whole gland primary therapy with curative intent for an organ confined prostate cancer, prostate specific antigen (PSA) < 20 ng/mL, Gleason score ≤ 7 (3 + 4), clinical stage T1N0M0-T2N0M0, and a follow-up longer than 12 months. Baseline physical examination and PSA measurements were obtained for all patients. Extracapsular tumor extension and lymph node status were also assessed for all patients using pelvic CT or MRI. Patients with incomplete oncologic data were excluded from the study.

Our technique of whole gland HIFU had been thoroughly described [12]. All patients were treated by a single experienced surgeon (RVV) with Ablatherm HIFU devices (EDAP-TMS, Vaulx-en-Velin, France). From September 2001 to March 2006, patients were treated with the first commercially available HIFU device from Ablatherm (Maxis-Technomed, Lyon, France) and since April 2006 with Ablatherm Integrated Imaging (Ablatherm, EDAP, Lyon, France). The same team (radiation therapist and physicist) and two experienced surgeons (Alexandre Peltier and Marc Vanden Bossche) performed LDR brachytherapy in the same years. All patients were treated by a permanent transperineal interstitial preloaded-free needles implantation of Iode^125^ using a real-time biplanar ultrasound-guided system. The postoperative dosimetric assessment was performed in all patients using computed tomography as recommended by the American Brachytherapy Society guidelines at one month of the implant which is considered essential for maintenance of a satisfactory quality assurance program [2]. In our institution the upper volume limit for HIFU and brachytherapy procedures is set to 40 cc and 50 cc, respectively. Patients with prostates exceeding this threshold are offered neoadjuvant cytoreductive androgen deprivation therapy (ADT). Hormonal treatment is always discontinued at the time of surgery.

### 2.1. Outcomes

Postoperatively, patients were followed with serial serum PSA determinations and digital rectal examinations at regular intervals. Oncologic outcomes were evaluated using the D'Amico tumor recurrence risk group classification system [13]. Biochemical recurrence rates were defined using the American Society for Therapeutic Radiology and Oncology (ASTRO)/Phoenix criteria (nadir + 2 ng/mL) and the Stuttgart criteria (nadir + 1.2 ng/mL) [14, 15].

Individual PSA nadir was identified in each patient. PSA nadir was defined as the lowest PSA value reached during follow-up. Cause of death was identified from patient file or from physician correspondence and all prostate cancer specific deaths were verified. Overall quality of life and costs were not reported in this study. The follow-up period was defined as the interval between surgery and last available monitoring data or the date of death. Complications were prospectively recorded and retrospectively graded according to the Clavien-Dindo score [16, 17]. Urinary functional outcomes were reported using physician reported rates. De novo or exacerbating postoperative LUTS were noted in the early setting and at long term of follow-up (>1 year). Stress incontinence was graduated according to Stamey into three grades [18]. Grade 1 was defined as loss of urine during heavy exercises, using not more than one pad per day, Grade 2 as loss of urine during light exercises but not at rest or during sleep, and Grade 3 as total loss of urine occurring at rest or during sleep. Patients that were able to penetrate their partner without mechanical or pharmacological support were rated potent.

### 2.2. Statistics

Patients treated by brachytherapy were matched 1 : 1 with patients undergoing whole gland HIFU in the same years. The matching procedure was blinded to the outcome in order to avoid selection bias. Matching criteria were in the following order: Gleason score, PSA, clinical tumor stage, D'Amico risk, and age. To confirm an appropriate matching, the absence of significant clinical and pathologic differences between the two cohorts of patients was assessed using Wilcoxon's rank-sum or *χ* ^2^ test, as appropriate. Similar analyses were conducted to investigate differences in perioperative and pathologic variables.

Univariate logistic regressions were performed to evaluate the impact of the surgical approach on complication occurrence. To evaluate possible amelioration of the technical aspects of the HIFU technique with the new device, we categorized the patients sequentially into two groups and analyzed the changes of the morbidity rate. Kaplan-Meier curves, log-rank test, and univariate Cox regression were constructed to analyze the influence of the surgical approach on recurrence-free survival, metastasis-free survival, cancer specific survival, and overall survival. A *p* value < 0.05 was considered to indicate statistical significance. A statistical analysis was performed with SPSS v. 20 (IBM Corp., Armonk, NY, USA).

## 3. Results

**Figure 1:**
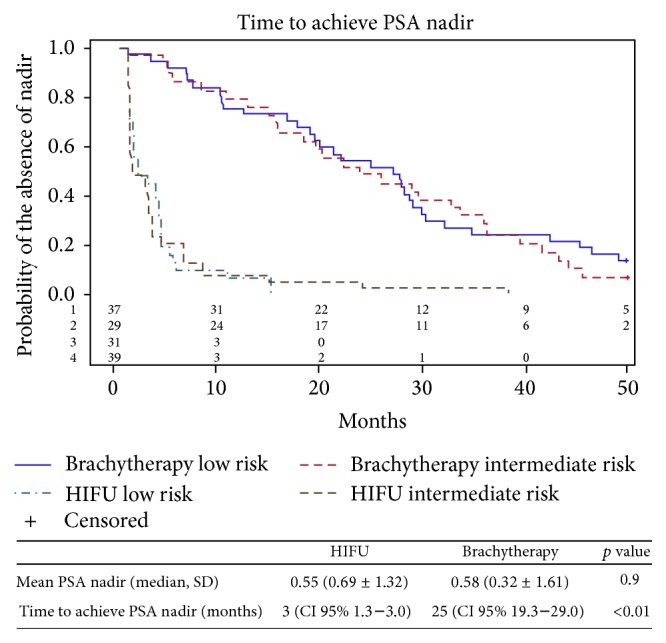
Time to achieve PSA nadir after HIFU and brachytherapy according to the classification of D'Amico.

**Figure 2:**
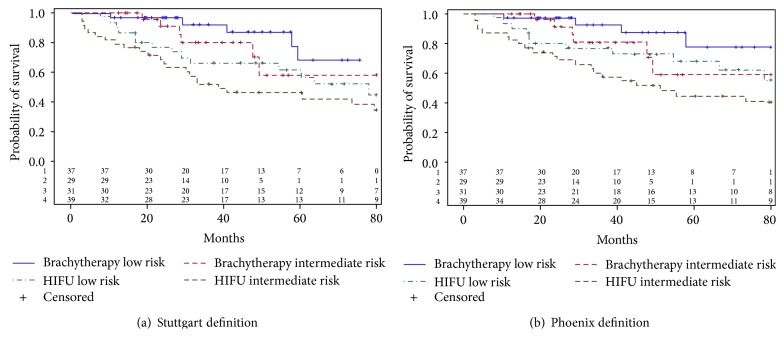
Kaplan-Meier curves for biochemical recurrence-free survival using Stuttgart (a) and Phoenix (b) definitions stratified according to D'Amico risk classification.

During the period of the study, 106 patients underwent LDR brachytherapy. Patients with incomplete oncologic data (4 patients) or limited follow-up < 12 months (32 patients) were excluded. A total of 70 patients have been included in the final analysis. These patients were matched with an equal number of patients treated by whole gland HIFU during the same years. Matching was successful with no statistically significant difference across the two groups except for the age (Table 1); patients operated by HIFU were older than patients undergoing brachytherapy (*p* < 0.01). The overall clinical and pathologic characteristics of the entire prospective HIFU cohort from which patients were selected for matching can be observed in Table 2. Median oncologic follow-up was statistically higher for the HIFU cohort compared to the brachytherapy cohort (83 months versus 44 months, *p* < 0.01). PSA nadir was noted in 95.7% of patients after HIFU and in 94.3% of patients after brachytherapy (Figure 1). The median time to achieve the nadir was statistically shorter in the HIFU cohort compared to the brachytherapy cohort (3 months versus 25 months, *p* < 0.05). Oncologic outcomes of the two cohorts are summarized in Table 3. The Phoenix and Stuttgart definitions were used for biochemical recurrence. Hazards ratio was calculated using HIFU cohort as a reference. The 5-year actuarial BRFS rates were significantly higher for the brachytherapy cohort compared to the HIFU cohort according to the Phoenix (68.5% versus 53%, HR = 0.41; CI 95%: 0.19–0.81, *p* < 0.05) and Stuttgart definitions (60.9% versus 53%, HR = 0.39; CI 95%: 0.19–0.74, *p* < 0.05), respectively. When stratifying patients according to the D'Amico risk and the technique used, BRFS rates were significantly higher for the low risk group treated by brachytherapy compared to the low risk group treated by HIFU according to Phoenix (77.5% versus 68%, HR = 0.31; CI 95%: 0.09–0.94, *p* = 0.05) and Stuttgart definitions (77.5% versus 56.3%, HR = 0.31; CI 95%: 0.10–0.84, *p* = 0.03), respectively (Figure 2).

**Figure 3:**
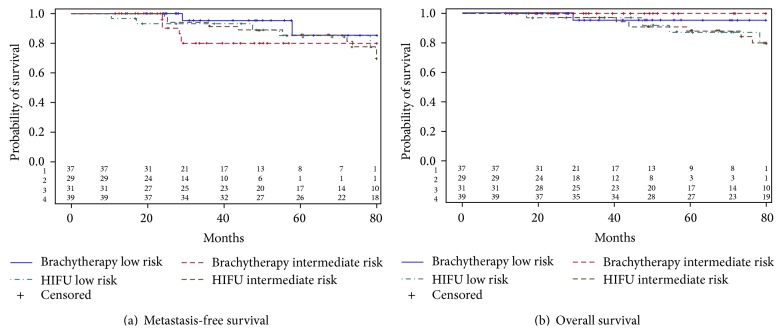
Kaplan-Meier curves for metastasis-free survival (a) and overall survival (b) stratified according to D'Amico risk classification.

For intermediate risk patients, there was no significant difference in BRFS rates between the brachytherapy and HIFU cohorts according to the Phoenix (58.8% versus 44.9%; HR = 0.47; CI 95%: 0.17–1.13, *p* = 0.12) and Stuttgart definitions (58.8% versus 42%; HR = 0.41; CI 95%: 0.15–0.97, *p* = 0.05), respectively. There was no significant difference in the 5-year actuarial metastasis-free survival rates (79.8% versus 85%, HR = 1.08; CI 95%: 0.36–2.95), cancer specific survival rates (92% versus 89%, HR = 0.67; CI 95%: 0.32–1.29), and overall survival (97.5% versus 88%; HR = 0.24; CI 95%: 0.01–1.34), for the brachytherapy and HIFU cohorts, respectively, even after stratifying according to the D'Amico risk (Figure 3).

The rates of the most common complications associated with these two procedures are reported in Table 4. HIFU cohort was divided into two subgroups according to the technique used. Urinary retention rates and urinary tract infection rates were significantly higher in the subgroup of patients treated with HIFU compared to brachytherapy. Lower urinary tract symptoms (LUTS) were the most frequent early and late postoperative complications with no statistically significant difference across the two cohorts. However, brachytherapy was associated with more storage and less voiding LUTS than HIFU. LUTS and hematuria were self-resolving in the majority of cases and their incidence decreased in the two groups after 1 year of follow-up. Only one patient in the brachytherapy arm had hemorrhagic cystitis in his follow-up managed by an endoscopic fulguration (Grade 3b). Gastrointestinal toxicity was low and comparable across the two cohorts with only one patient in each group developing a rectourethral fistula managed surgically (Grade 3b). Bladder outlet obstructions mainly urethral stricture and chronic pelvic pain were encountered more frequently following HIFU in particular in patients treated early with the first commercially available HIFU device. Finally, no peri- or postoperative deaths were recorded in the two cohorts (Table 5).

Regarding urinary incontinence, no significant difference was found across the two cohorts (7.2% versus 3.8%, *p* = 0.44). Transient urinary incontinence was seen in two patients one in each group. The only patient with persistent incontinence at the last follow-up in the brachytherapy group had a mixed incontinence. This patient had already been treated by optical urethrotomy. In the HIFU cohort, Grade 1 persistent stress urinary incontinence was reported in 2 patients and the 2 other patients had Grade 2 stress urinary incontinence. In preoperatively potent patients in the HIFU cohort (*n* = 43), 5 men (11.6%) had documented post-whole gland ablation erectile dysfunction (ED) and 27 men (62.8%) had erections satisfactory for sexual intercourse with (*n* = 9) or without pharmacotherapy (*n* = 20), and data were lacking in 9 patients. For the brachytherapy group, data on preoperative erectile function were not available for the majority of patients.

## 4. Discussion

The aim of this study was to compare retrospectively HIFU and brachytherapy for the curative treatment of organ confined prostate cancer.

The two techniques are effective as demonstrated by the high probability to achieve low PSA nadir following treatment. However, the early achievement of PSA nadir following HIFU compared to brachytherapy provides an immediate feedback on treatment efficacy and identifies quickly patients with residual cancer. This rapid proof of response provides also stringent information about potential cure as demonstrated in most contemporary series [19–21]. In our previous study, only 12.5% of patients achieving PSA nadir < 0.5 ng/mL experienced biochemical failure at 10 years of follow-up [12].

In our clinical practice, assessment of oncologic efficacy is performed by serial PSA testing and random systematic TRUS guided biopsies are offered only for a cause (Phoenix criteria and/or suspicious DRE) in order to minimise burden on the patient. Moreover, systematic post-HIFU biopsy mapping of treated prostates has widely proven the local efficacy of thermoablation. Although PSA testing is accepted as a valid outcome to define biochemical recurrence after brachytherapy, its clinical significance following tissue ablation is not yet determined [22]. To date, studies reporting on HIFU are using the Phoenix definition or the more recent Stuttgart definition to define biochemical recurrence but these two definitions are not validated for the HIFU group. Of note it has to be reminded that HIFU technical procedures leave untreated significant areas of the prostate, that is, up to 7 mm of apical area and anterior sectors of the prostate beyond 26 mm measured from the posterior capsule.

Our results for the two cohorts are in line with the reported rates published in the contemporary literature that had used a combination of biopsy results and PSA threshold values to assess failure [23]. According to these criteria, BRFS was significantly higher for the low risk group following brachytherapy compared to HIFU. This could be explained by the absence of matching for year of treatment across our two groups that resulted in a significantly longer follow-up in the HIFU cohort. The longer follow-up in the HIFU cohort may lead to the increased number of biochemical recurrences detected in this group. As such, this bias should have also favoured brachytherapy for the intermediate risk. One could argue that the absence of difference is due to an early biochemical recurrence in the intermediate group treated by brachytherapy or even a worse prognosis. In the literature, there has been much discussion on the relative impact of Gleason score 7 on oncologic outcome following treatment by brachytherapy [24–27]. The majority of reports on outcomes after brachytherapy show inferior biochemical control for patients with Gleason 7 [28, 29]. The EAU and ABS exclude patients with a Gleason 7 from the qualification criteria for a brachytherapy alone treatment [2, 30]. For the NCCN, brachytherapy is always combined to external beam radiation therapy (EBRT) for intermediate risk patients [31]. A new study had suggested the need for high quality and high-dose treatment to eradicate Gleason 7 prostate cancer [32]. At present, there is an ongoing phase III prospective randomized controlled open label registered clinical trial (http://www.clinicaltrials.gov/ Identifier: NCT00063882). The trial randomizes patients with intermediate-risk prostate cancer to brachytherapy alone versus combined therapy (brachytherapy and extended beam radiation therapy) in order to define the optimum brachytherapy treatment for patients with intermediate-risk prostate cancer. In the early experience with HIFU, there was much disagreement on treating patients with high risk disease. Some authors have argued that HIFU is a coagulative technology that unlike radiation therapy results in a complete cell destruction independently of Gleason score. However, results have clearly demonstrated that tissue under ablation in high grade tumour may be inadequately ablated (heat sinks phenomenon) and is a high risk site for persistent residual progressive disease and metastatic spread [12, 33]. For patients with intermediate risk of progression, results are contradictory and the debate is ongoing [12]. The main advantage of HIFU over brachytherapy is the possibility to repeat the treatment with a further increase in response rate without substantially increasing side effects [34]. It is also noteworthy to mention that, by using the stricter Stuttgart definition, studies with a short follow-up will experience more failures but possibly achieved a truer picture of outcomes and allow earlier treatment of patients. However, with longer follow-up, the difference between the two criteria will be reduced which is demonstrated in the present study by the small percentages of patients considered as failures according to Stuttgart but not to Phoenix definition. The 5-year actuarial metastasis-free survival, cancer specific survival, and overall survival rates were not significantly different between the two cohorts and are in line with the reported rates in nonrandomized published case series [35]. However, the retrospective design and the insufficient follow-up limit further conclusion.

Early side effects and complications of the two techniques involve primarily the urinary tract. Storage and voiding LUTS develop immediately as a result of implant trauma after brachytherapy or tissue heating following HIFU. Over the first weeks LUTS are due to the effect of radiation and tissue sloughing and necrosis following brachytherapy and HIFU, respectively. At 3 months, HIFU was more associated with voiding LUTS whereas brachytherapy was more associated with storage LUTS. These urinary symptoms are generally mild and self-resolving after several months as evidenced by the lower rate at the last follow-up. Technical improvements with the introduction of the new Ablatherm device (frequency of the ultrasound, length of treatment pulses, and rectal cooling) and the changes in surgical protocol (concomitant TURP and prophylactic antibiotics) had substantially lowered the high rate of urinary tract infection and bladder outlet obstruction encountered at our early experience with HIFU. One patient developed rectourethral fistula in each group.

The incidence of this devastating complication following brachytherapy and HIFU had substantially decreased over the last decade [36]. This is not only due to technical improvements but also to a better understanding of the management of postoperative rectal bleeding. Regarding urinary incontinence, no significant difference was found across the two cohorts. The incidence of urinary incontinence after prostate brachytherapy varies between 0% and 12.9% [37, 38]. The study with the longest follow-up reported a 5.1% risk of urinary incontinence [39]. This result was confirmed by Benoit et al. who estimated the incidence of urinary incontinence to be 6.6% in a study of 2,124 men in a population of Medicare patients with a follow-up from 2 to 3 years [40]. The mechanism is partially elucidated and studies reporting correlation between urinary incontinence and dosimetric or clinical parameters had been published [41]. The incidence of incontinence is also low after HIFU with reported rates varying between 5 and 11% [42]. Safety margins at the level of the apex of the prostate are calculated to avoid temperature diffusion and lesions to the urethra and striated sphincter. The same precautions are taken laterally, to avoid causing lesions to the neurovascular bundles in order to preserve erectile function. These precautions should always be balanced with the risk of oncologic failure [43]. In the HIFU cohort, if we consider patients with lacking data to have ED, the rate after treatment would be 32.5% which is in the range of reported rates in the literature [44]. Recently, we have reported better stress urinary incontinence and erectile function rates with hemiablation HIFU but validated questionnaire and further experience are needed to confirm these findings [45]. Unfortunately, the erectile function data for patients treated by brachytherapy are unavailable. To our knowledge, our study is the first to compare patients treated by HIFU and brachytherapy. However, we acknowledge the limitations of this retrospective study. Physician reported rates and physician-acquired information have been shown to correlate poorly with data collected from patient self-assessment questionnaires. In addition, the study reported retrospectively on a small cohort of patients. Well-designed, multicenter, prospective, randomized controlled studies are required to assess collateral damage and functional and oncologic outcomes. Another limitation of the present study is the absence of matching for the year of treatment and for the follow-up across the two groups which was already discussed.

## 5. Conclusion

HIFU and brachytherapy are two minimal invasive safe options for the treatment of organ confined prostate cancer which produce different short term complications but similar long term functional outcomes. Regarding oncologic outcomes, our data reported similar 5-year metastasis-free survival, cancer specific survival, and overall survival in both cohorts of patients. Well-designed, multicenter, prospective, randomized controlled studies with a higher number of patients and a longer follow-up are needed to confirm these results.

## Figures and Tables

**Table 1:** Baseline patient characteristics after matching.

	Brachytherapy (*n* = 70)	HIFU (*n* = 70)	*p* value
Median age (IQR; SD), years	69 (54–79; 6.5)	74 (62–86; 4.47)	<0.01
Clinical stage (T)			0.54
T1a	1	2	
T1b	2	6	
T1c	38	31	
T2a	20	19	
T2b	9	12	
Gleason score			1
≤6	51	51	
7	19	19	
PSA (ng/mL)			0.23
≤10	57	50	
>10 et ≤20	13	20	
D'Amico risk classification			0.33
Low	33	31	
Intermediate	37	39	
Neoadjuvant ADT	14	19	0.43
ASA score			0.68
1	8	10	
2	47	42	
3	15	18	
Median follow-up (IQR), months	44 (21–70)	83 (29–98)	<0.01

**Table 2:** Baseline and tumour characteristics of 110 patients with localized prostate cancer who were treated by a single session of high intensity focused ultrasound.

Mean age, years [range]	76.1 ± 6.2 [61–86]
Mean preoperative PSA, ng/mL [range]	12.1 ± 4.1 [0.55–49.0]
Mean prostate volume, mL [range]	29.3 ± 6.0 [18–39]
Hormone, *n* (%)	
Yes	37 (33.6)
No	73 (66.4)
Gleason score, *n* (%)	
≤6	69 (62.7)
7	24 (21.8)
≥8	17 (15.5)
Stage, *n* (%)	
T1	51 (46.4)
T2	59 (53.6)
D'Amico risk group^**∗**^, *N* (%)	
Low	40 (36.4)
Intermediate	49 (44.5)
High	21 (19.1)

^**∗**^Risk group based on D'Amico definition (according to Stage, Gleason, and PSA).

**Table 3:** Oncologic outcomes for the cohort stratified according to the D'Amico risk classification.

	Brachytherapy	HIFU^*∗*^	Hazards ratio (CI^*∗∗*^ 95%)
*Biochemical recurrence-free survival rates *	Phoenix definition: 68.5%	Phoenix definition: 53.1%	0.41 (0.19–0.81)
	Stuttgart definition: 60.9%	Stuttgart definition: 51.3%	0.39 (0.19–0.74)
Low risk	Phoenix definition: 77.5%	Phoenix definition: 68%	0.31 (0.09–0.94)
	Stuttgart definition: 77.5%	Stuttgart definition: 56.3%	0.31 (0.10–0.84)
Intermediate risk	Phoenix definition: 58.8%	Phoenix definition: 44.9%	0.47 (0.17–1.13)
	Stuttgart definition: 58.8%	Stuttgart definition: 42%	0.41 (0.15–0.97)
*Metastasis-free survival rates*	79.8%	85%	1.08 (0.36–2.95)
*Cancer specific survival rates*	92%	89%	0.67 (0.32–1.29)
*Overall survival rates*	97.5%	88%	0.24 (0.01–1.34)

^*∗*^HIFU: high intensity focused ultrasound; ^*∗∗*^CI: confidence interval.

**Table 4:** Comparison of early and long term postoperative complications according to the therapeutic approach.

	BT^*∗*^	HIFU^*∗∗*^	Maxis Ablatherm	Ablatherm integrated imaging	*p* value (BT versus HIFU)	*p* value (BT versus Maxis)	*p* value (BT versus integrated imaging)
(A) Early							
*N* =	59	90	39	31	—	—	—
Acute urinary retention	4 (6.8%)	16 (22.9%)	12 (30.8%)	4 (12.9%)	0.02	<0.01	0.44
Urinary tract infection	5 (8.5%)	15 (21.4%)	12 (30.8%)	3 (9.7%)	0.07	<0.01	0.86
LUTS^*∗∗∗*^	25 (42.4%)	28 (40.0%)	17 (43.6%)	11 (35.5%)	0.93	1	0.68
(i) Storage	19 (32.2%)	5 (7.2%)	3 (7.7%)	2 (6.4%)	<0.01	<0.01	0.01
(ii) Voiding	6 (10.2%)	23 (32.8%)	14 (35.9%)	9 (29.1%)	<0.01	<0.01	0.04
Gastrointestinal toxicity	2 (3.4%)	1 (1.4%)	1 (2.6%)	0 (0.0%)	0.59	1	0.78

(B) Long term							
*N* =	53	69	39	30	—	—	—
LUTS^*∗∗∗*^	14 (26.4%)	19 (27.5%)	12 (30.8%)	7 (23.3%)	1	0.82	0.96
(i) Storage	10 (18.9%)	7 (10.1%)	4 (10.3%)	3 (10.0%)	0.26	0.30	0.36
(ii) Voiding	4 (7.5%)	12 (17.4%)	8 (20.5%)	4 (13.3%)	0.18	0.18	0.45
Urethral stricture	2 (3.8%)	17 (24.6%)	12 (30.8%)	5 (16.7%)	<0.01	<0.01	0.09
Rectourethral fistula	1 (1.9%)	1 (1.4%)	1 (2.6%)	0 (0.0%)	1	1	1
Chronic pelvic pain	0 (0.0%)	3 (4.3%)	3 (7.7%)	0 (0.0%)	0.34	0.08	1
Urinary incontinence	2 (3.8%)	5 (7.2%)	3 (7.7%)	2 (6.7%)	0.44	0.72	0.62

^*∗*^Brachytherapy, ^*∗∗*^high intensity focused ultrasound, and ^*∗∗∗*^lower urinary tract symptoms.

**Table 5:** Comparison of overall complications by grade of severity between the two groups.

	Grade 1		Grade 2		Grade 3a		Grade 3b	
	HIFU	BT	HIFU	BT	HIFU	BT	HIFU	BT
LUTS/hematuria (early)	23/70 (32.9%)	18/59 (30.5%)	5/70 (7.1%)	7/59 (11.9%)	0 (0%)	0 (0%)	0 (0%)	0 (0%)
LUTS/hematuria (late)	3/69 (4.3%)	2/53 (3.8%)	16/69 (23.2%)	11/53 (20.7%)	0 (0%)	1/53 (1.9%)	0 (0%)	0 (0%)
Urinary tract infection	0 (0%)	0 (0%)	15/70 (21.4%)	5/59 (8.5%)	0 (0%)	0 (0%)	0 (0%)	0 (0%)
Acute urinary retention	0 (0%)	0 (0%)	0 (0%)	0 (0%)	14/70 (20.0%)	4/59 (6.8%)	2/70 (2.8%)	0 (0%)
Urinary stricture	0 (0%)	0 (0%)	0 (0%)	0 (0%)	2/69 (2.9%)	0 (0%)	15/69 (21.7%)	2/53 (3.8%)
Urinary incontinence	5/69 (7.2%)	1/53 (1.9%)	0 (0%)	1/53 (1.9%)	0 (0%)	0 (0%)	0 (0%)	0 (0%)
Gastrointestinal toxicities	0 (0%)	0 (0%)	0 (0%)	1/59 (1.7%)	0 (0%)	0 (0%)	1/70 (1.4%)	1/59 (1.7%)
